# Dental Work in the Press

**Published:** 1842-12

**Authors:** 


### Dental Work in the Press.

*Maury's Dental Surgery.*—We announced in the last number of our second volume, that Dr. J. B. Savier was engaged in the translation of the above work; and we now have the pleasure of informing our readers that it is passing through the press of the enterprising and extensive medical publishers, Messrs. Lea & Blanchard, of Philadelphia, so that the members of the profession will very soon have it in their power of adding this valuable treatise, in an English dress, to their libraries.

Of the merits of the work, we have already expressed our opinion. In France it ranks among the very first upon the practice of dental surgery; and although the author advances many opinions which we conceive to be incorrect, we can, notwithstanding, recommend the work as one among the best on the subjects on which it treats. But, as we may have occasion to speak of the work hereafter, it is not necessary to say more at present, than merely to direct the attention of the profession to it.

